# Laterally Resolved Free Energy Profiles and Vibrational Spectra of Chemisorbed H Atoms on Pt(111)

**DOI:** 10.1021/acs.jctc.3c00997

**Published:** 2024-02-07

**Authors:** Sudarsan Surendralal, Mira Todorova, Jörg Neugebauer

**Affiliations:** Department of Computational Materials Design, Max-Planck-Institut für Eisenforschung GmbH, Max-Planck-Straße 1, Düsseldorf D-40237, Germany

## Abstract

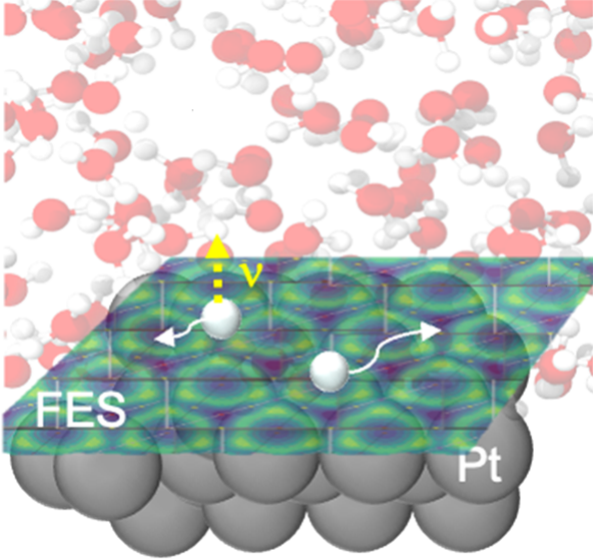

A scheme to compute laterally resolved free energy surfaces and spectral signatures of specifically adsorbed ions on electrode surfaces from their *ab initio* molecular dynamics (AIMD) trajectories is proposed. Considering H-covered Pt(111) electrodes, both in contact with water and vacuum and for various H coverages, we systematically explore the impact of explicit water and H-coverage on site occupancy, providing direct insight into the proportion of underpotential and overpotential deposited hydrogen adsorbates. Extending this approach further, we can obtain laterally resolved vibrational spectra of the Pt–H stretch modes. We discuss how the difference between the free energy basins of the on-top and fcc-hollow adsorption sites explains the features of the experimentally observed spectral fingerprints in this system. These fingerprints do not contain only information about the stable and metastable adsorption sites but also about intermediate short-lived adsorbate configurations. Our results also show that for these properties chemisorbed H_2_O acts as a spectator and does not qualitatively influence the relative stabilities of the adsorption sites and their spectral fingerprints.

## 1 Introduction

The kinetics of electrochemical reactions is often controlled by specifically adsorbed ions on electrode–electrolyte interfaces. In particular, the nature of hydrogen adsorption on transition metal electrodes plays an important role in the hydrogen evolution reaction (HER), which is the cathodic step of electrochemical water splitting. For this reason, hydrogen adsorption on such metal electrodes, particularly platinum, has been widely investigated using experimental techniques such as cyclic-voltammetry,^1−3^ as well as using *ab initio* computational modeling techniques like density functional theory (DFT).^4−12^

A fundamental challenge in understanding the role hydrogen adsorbates play in electrochemical reactions is to precisely determine the sites on which they are adsorbed under given electrochemical conditions. A prominent example is the Pt(111)/H_2_O interface, which is one of the most extensively studied prototypical electrochemical interfaces.^2,4,5,10,12−25^ Hydrogen adsorption on Pt(111) can, in principle, occur on four high-symmetry sites: singly coordinated on-top adsorption sites, doubly coordinated bridge adsorption sites, 3-fold coordinated hollow fcc sites, and 3-fold coordinated hollow hcp sites.

In electrochemistry, specifically adsorbed hydrogen atoms (H_ad_) are classified based on the potentials at which they are adsorbed on the surface of the electrode. Underpotential deposited hydrogen, H_UPD_, refers to the hydrogen adsorbed above the equilibrium potential (*i.e.*, at potentials less negative than 0 V *vs* the reversible hydrogen electrode—RHE) and overpotential deposited hydrogen H_OPD_ is the hydrogen adsorbed below this potential.^8,25^ It is generally assumed that H_UPD_ binds more strongly to the electrode than H_OPD_ and that H_OPD_ dominates the HER or the hydrogen adsorption/desorption reaction (H_ad_ ↔ H_aq_^+^ + e^–^), while H_UPD_ is a mere spectator.^8,25^ Due to the fundamental nature of this question, enormous effort has been devoted to understanding the relative stability of these two configurations of hydrogen adsorbates and how they change with varying pH and/or electrode potential.

Experimental studies focusing on the Pt(111) electrode have assigned H_UPD_ to the fcc-hollow adsorption sites^26^ and H_OPD_ to the on-top adsorption sites,^27^ although there is still an ongoing controversy about this assignment.^23,28^ Studies using static DFT calculations have found the fcc hollow site to be the energetically most stable site.^11,12,25^ However, since the energy differences between the adsorption sites are small and hydrogen adsorbates are highly dynamic even at room temperature, these adsorbate structures have in reality most likely a mixed occupation of hollow and on-top adsorption sites. There is also some uncertainty regarding the observed spectroscopic signatures of the adsorbates. Experimental and theoretical spectroscopic studies have assigned the broad peak at 1100 cm^–1^ to the hollow fcc adsorption site^7,11^ and the peak at 2200 cm^–1^ to the weakly bound on-top adsorption site.^7,11,29^ Results from AIMD based computational studies,^22,23^ however, also indicate the presence of a bridge site intermediate which is expected to correspond to a frequency of ≈1050 cm^–1^.^29,30^

To address these issues, we compute in this work (i) laterally resolved free energy surfaces and (ii) laterally (*i.e.*, adsorbate site) resolved vibrational spectra of H on Pt(111). In the following, we first describe the methodology to construct these from state-of-the-art DFT-MD simulations. We then use these approaches to systematically study the impact of H coverage and the presence/absence of H_2_O on free energy and spectroscopic signatures. The presented methodology relies on only the equilibrated molecular dynamics trajectories of the hydrogen adsorbates as input. These results help not only to understand how the relative stability of the different sites varies with H coverage and the presence of solvent but also to identify the microscopic origin of experimentally observed spectroscopic signatures.

## 2 Computational Details

**Figure 1:**
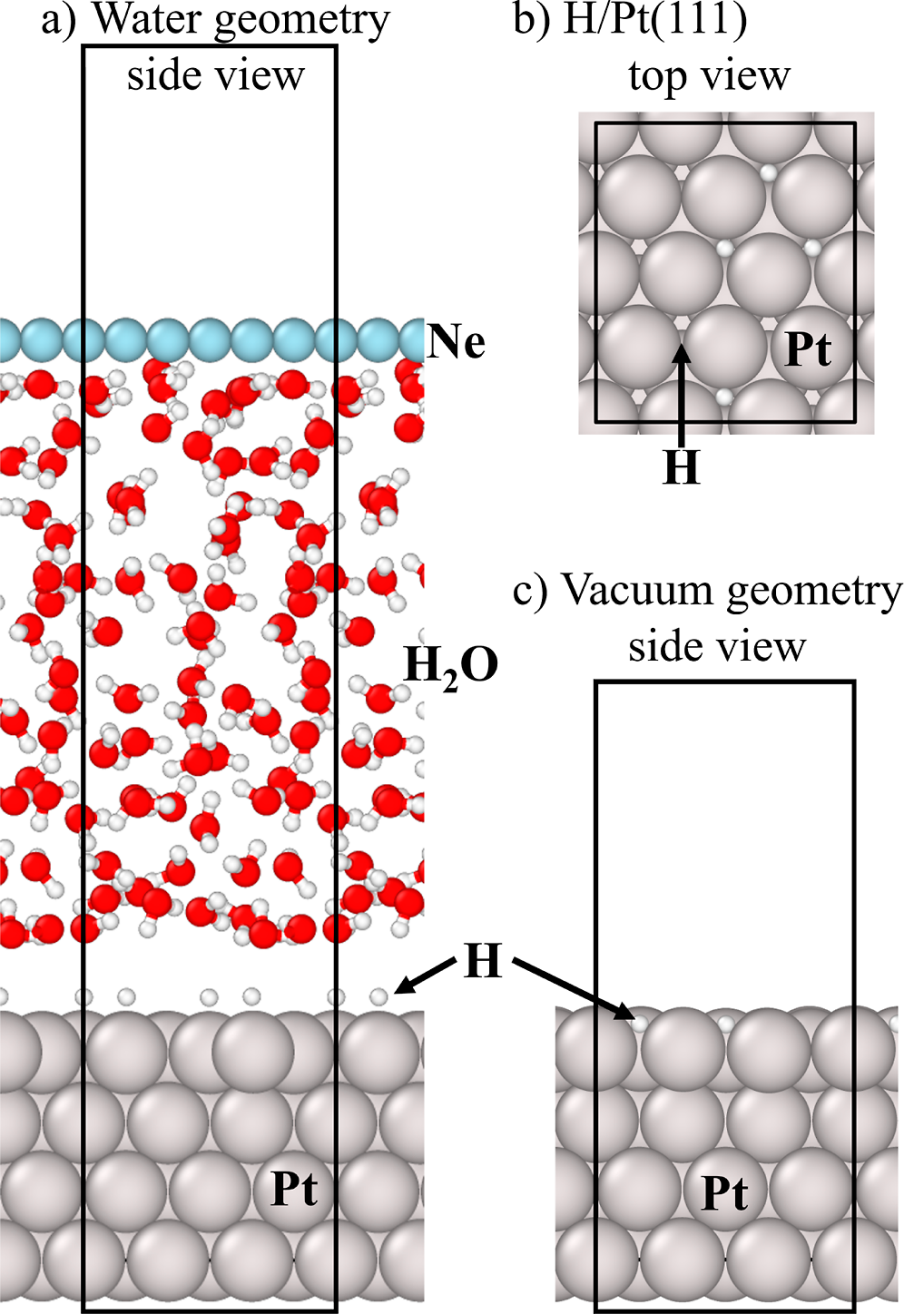
Representative snapshots of periodic supercells used for the simulations of the Pt(111)–H systems showing a side view of the simulation cell (*i.e.*, along the surface normal) in (a) with the presence of water and (c) without the presence of water, and in (b) a top view of the H/P(111) surface. The gray, red, white and cyan spheres represent the Pt, O, H and Ne atoms, respectively. The dark black boxes represent the periodic boundaries of the supercells. The Ne “counter electrode” in (a), which was introduced in our previous work,^6,32^ is “uncharged” and used to maintain the density of water in the simulation.

**Figure 2:**
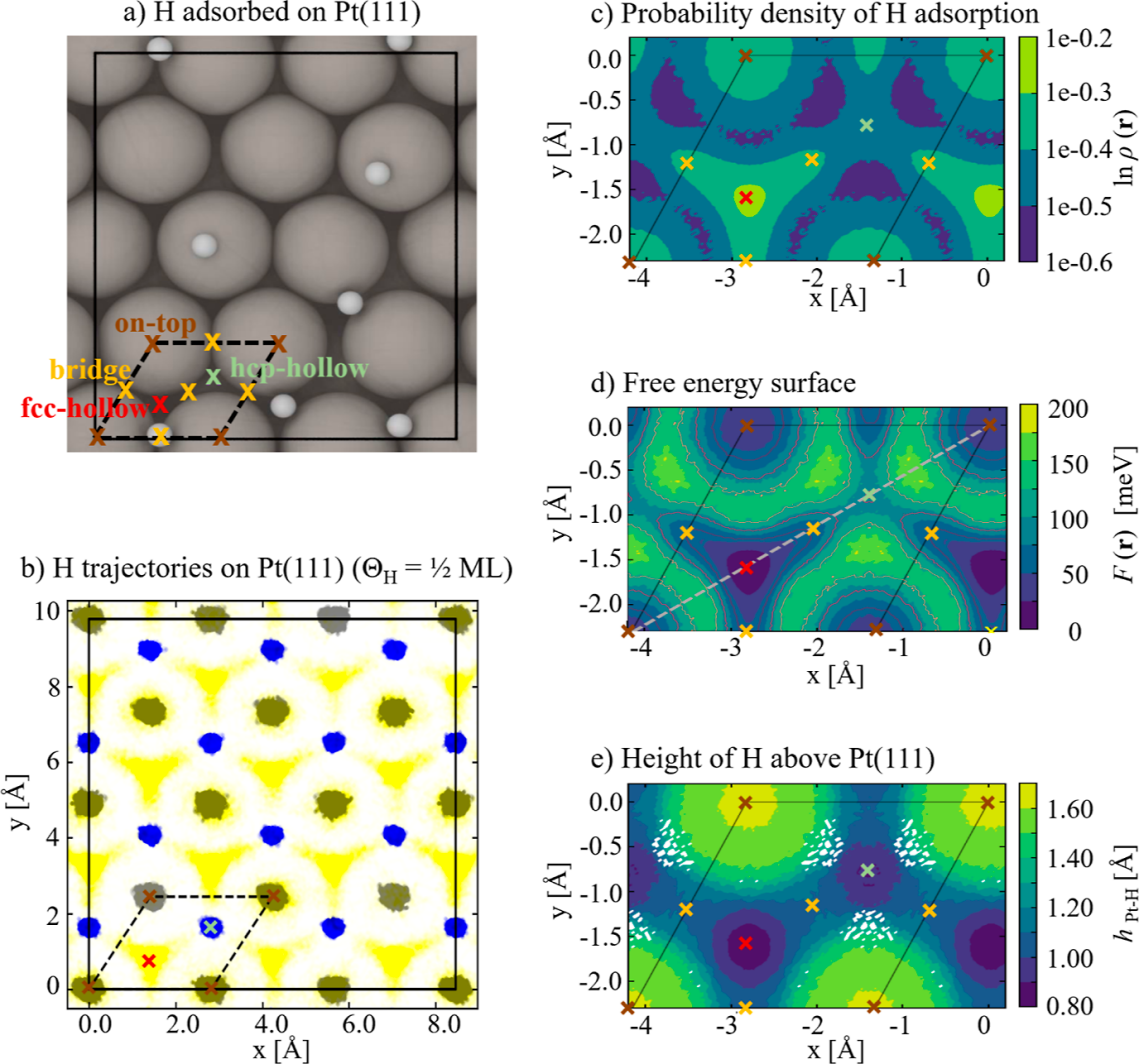
(a) Top view of a representative snapshot of H adsorbates on the  rectangular surface cell of Pt(111) taken from the DFT-MD simulations for Θ_H_ = 1/2 ML coverage (in the absence of water). The dashed and solid black boxes shown in (a,b) represent the periodic boundaries of the (1 × 1) surface unit cell and the periodic boundaries for the  rectangular surface cell, respectively. The high symmetry adsorption sites are shown in all figures as colored crosses. (b) Point cloud of the projected two-dimensional trajectories of the hydrogen adsorbates (in yellow) and the surface (in green) and subsurface (in blue) Pt atoms, visualizing their thermal motion. (c) The two-dimensional density ρ(***r***_∥_) of hydrogen adsorption on Pt(111). (d) Corresponding free energy profile *F*(***r***_∥_) obtained from eq 2. The purple, magenta, and light-orange contour lines correspond to free energy values of 60, 90, and 120 meV (see text). (e) The averaged height of hydrogen adsorbates above the Pt surface at each grid point on this surface. The (1 × 1) surface unit cell is shown by the black parallelogram in (c–e).

The Pt(111) electrode is modeled as 4 monolayers of a  rectangular surface cell (with 12 Pt atoms per layer), as seen in Figure 1. Hydrogen atoms corresponding to hydrogen coverages Θ_H_ ranging from 1/12 ML to 1/2 ML (1–6 H atoms) are placed on top of the electrode (Figure 2a). Two sets of DFT-MD calculations, with and without water, are then performed. For the calculations in the presence of H_2_O, 64 water molecules (pre-equilibrated using classical molecular dynamics with the TIP3P water potential^31^) are sandwiched between a Pt electrode and a Ne counter electrode based on the scheme described in our previous work.^6,32^ The Ne counter electrode is followed by a vacuum region of 12 Å width to decouple the two sides of the slab system (Figure 1). The adsorbates and the first two Pt layers facing the solvent are allowed to relax, while the other two Pt layers facing vacuum and the Ne electrode atoms are fixed.

All DFT calculations are performed using the Vienna *ab initio* simulation package (VASP)^33−35^ with the projector augmented wave method.^36^ Based on the recommendation by Sakong and co-workers,^37^ the revised PBE (RPBE) functional^38,39^ together with D3 type dispersion corrections^40^ are used. A plane-wave energy cutoff of 500 eV is used to expand the wave functions, along with a Fermi-type electronic occupancy smearing width of 0.1 eV. A reciprocal space (*k*-point) mesh density of (2 × 2 × 1) using the Monkhorst–Pack scheme^41^ is employed. The DFT-MD calculations are performed in the canonical ensemble at a target temperature of 300 K using a time step of 0.9 fs and a Langevin thermostat. The entire workflow for the calculations described in this work is automated using the pyiron package.^42^

The DFT-MD simulations are run for 150 ps (after 20 ps of equilibration) for the Pt(111)/H/vacuum systems and for 20–30 ps (after 5 ps of equilibration) for the Pt(111)/H/H_2_O systems, which are computationally much more expensive. We note that the convergence of the quantities calculated in this work is dependent on the total simulation time since longer simulation times allow for a better sampling of all possible adsorption sites.

For the system with H_2_O, at some of the higher H-coverages a single H_ad_ desorbs from the Pt surface to form a solvated proton. Since this desorption changes the surface coverage, the trajectories of the system before and after desorption are considered separately.

## 3 Computing Free Energy Profiles

To compute the laterally resolved free energy surface and vibrational spectra, we consider first a specific example: the Θ_H_ = 1/2 ML interface without any solvent. The two-dimensional lateral trajectories (for the surface plane parallel to the electrode surface)  of the H atoms adsorbed on the electrode surface are plotted in Figure 2b. The resulting trajectories extend over a broad region, highlighting the dynamic character of H adsorbates, even at room temperature. The figure also shows two very different local distributions: An isotropic (spherically) shaped one centered at the on-top site and a tristar shaped one with the center at the fcc-hollow adsorption site. Thus, at room temperature only two (meta)stable adsorption sites (on-top and fcc-hollow) exist.

While the H trajectories projected on the 2D surface provide already some important insights, a physically much richer quantity is the lateral density of hydrogen adsorption (Figure 2c)
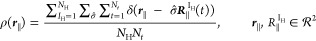
1

The lateral density ρ(***r***_∥_) gives the probability to find one of the adsorbates at the lateral position ***r***_∥_. Here, δ is the Dirac delta function which is used to construct a two-dimensional histogram of the lateral positions of the H adsorbates on a grid spanning the primitive Pt(111) surface unit cell represented by ***r***_∥_. The sums run over all hydrogen atoms (index *I*_H_) with *N*_H_ being the total number of H adsorbates in the system (*N*_H_ = 6 in this example), *N*_*t*_ being the number of the DFT-MD time steps (with index *t*), and the point group and translational symmetry operators of the Pt(111) surface . For the here considered fcc (111) surface, the 3-fold *C*_3*V*_ symmetry applies. Including both translational and rotational symmetries is a key feature of this approach and boosts the number of data points by more than 2 orders of magnitude, providing statistically well converged densities already at rather modest MD simulation times (*cf.*Figure 2c).

Assuming thermodynamic equilibrium for the canonical MD ensemble, the two-dimensional Helmholtz free energy profile of the adsorbates on the electrode surface can be computed by the Boltzmann inverse of the computed lateral density ρ(***r***_∥_)^43^
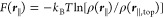
2with *k*_B_ being the Boltzmann constant, *T* the temperature and ***r***_∥,top_ the lateral position of the on-top side. This formula ensures that the free energy minimum at the fcc-hollow adsorption site is 0 eV, allowing us to straightforwardly determine the relative free energy difference of hydrogen adsorption at different adsorption sites. The free energy surface for the case with 1/2 ML of H is shown in Figure 2d. It reveals the presence of two symmetry-inequivalent free energy basins: one corresponding to the fcc-hollow adsorption site and the other corresponding to the on-top one.

**Figure 3:**
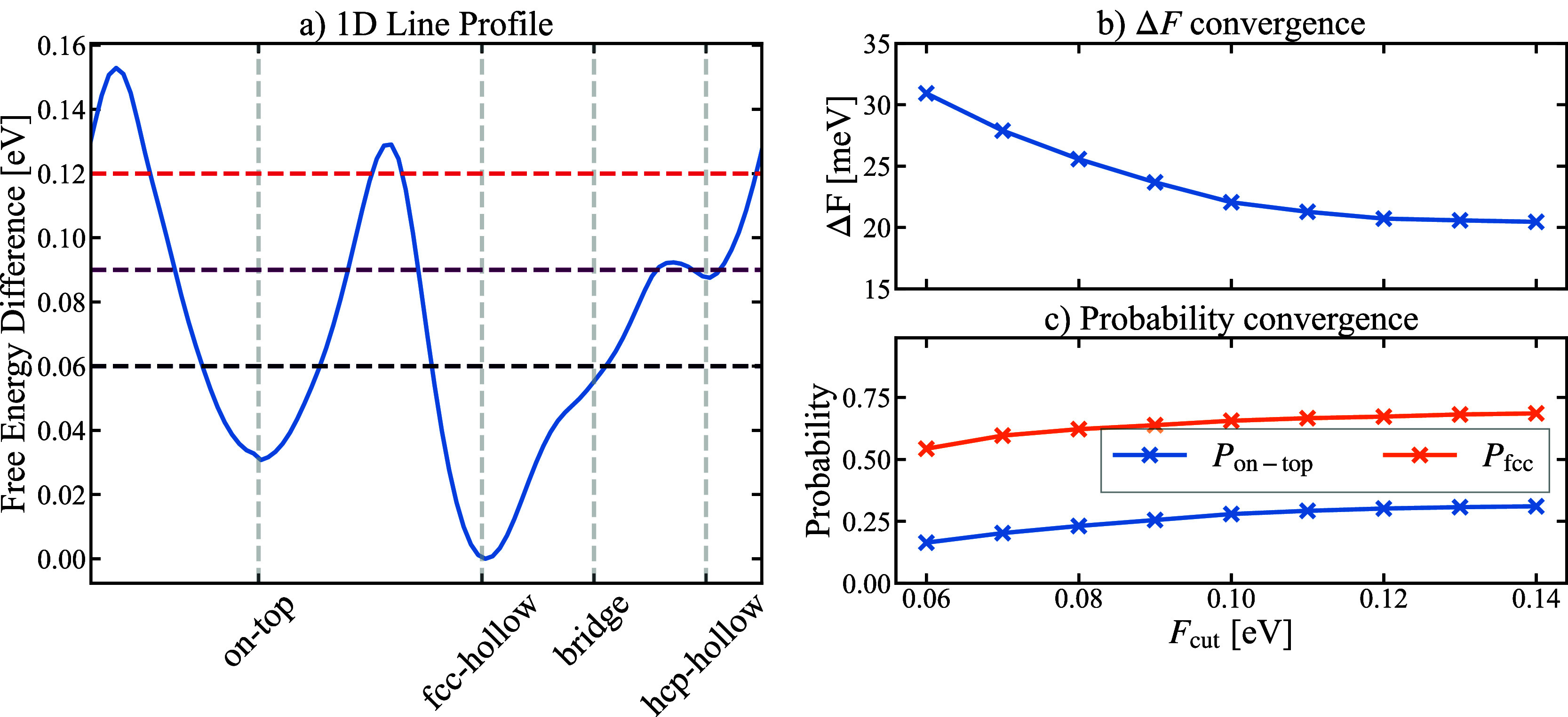
(a) The line profile of the free energy difference for Θ_H_ = 1/2 ML (with respect to the minima at the fcc-hollow site) obtained when traversing the different adsorption sites along the diagonal (gray dashed line in Figure 2d) of *F*(***r***_∥_). A Gaussian type smoothening is applied to this profile. The values are aligned with respect to the free energy at the fcc-hollow adsorption site (energy zero). (b) Convergence of the free energy differences and (c) site occupancy probabilities (see text) as a function of the energy cutoff (*F*_cut_).

The corresponding line profile of the free energy when traversing across the diagonal (indicated by the dashed line in Figure 2d) of the (1 × 1) Pt(111) unit cell is shown in Figure 3a. The fcc-adsorption site is the most stable one, followed by the on-top adsorption site. The free energy around the bridge adsorption site shows no local minimum, *i.e.*, this site is therefore not a metastable configuration as suggested by a few studies in the past,^23^ but is part of the extended fcc-hollow basin. The hcp-hollow site is the least stable of all adsorption sites.

The determination of the energy difference between two adsorption sites at *T* = 0 K is straightforward since the lateral position of the H atom is well-defined by the surface geometry and symmetry, with a distribution given in the classical limit by the infinitely sharp Dirac function. At finite temperatures, however, the H distribution is smeared out over the entire surface. Thus, to determine the probability of finding a H atom at one or the other adsorption site requires to define lateral regions which belong to one or the other adsorption site.

Conceptually, this task is similar to decomposing the total charge density on the individual atoms. Similar to the charge density decomposition, there is no single unique approach that exists but rather several physically motivated and pragmatic approaches. In the following, we outline our strategy, which is based on decomposing the free energy surface into individual basins by using an energy cutoff.

Starting point of our approach is the relative free energy difference between two basins that can be determined by
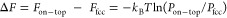
3

Here, *F*_on-top_ and *F*_fcc_ are the free energies of the on-top and fcc-hollow basins, while *P*_on-top_ and *P*_fcc_ represent the corresponding total probabilities of finding an adsorbate in one of these basins. To determine these probabilities, we first separate the two free energy basins by defining a free energy cutoff *F*_cut_. Only spatial regions with *F*(***r***) ≤ *F*_cut_ values are considered to be part of either basin. To identify all positions ***r*** fulfilling the above condition and belonging to the same energy basin (*i.e.*, either to the one corresponding to the top or to the hollow adsorption site) we apply a *K*-means clustering algorithm. As neighbor distance in the clustering algorithm we chose the distance between two neighboring points of the numerical grid on which the H density is projected. As input features to the clustering approach, we use the adsorbate height rather than the energy surface. The height profile turned out to be smoother and more sensitive with respect to the adsorbate site compared to the energy surface. In addition, the distance of the grid points to the center of each of the two basins was given as input to the clustering approach.

This combined approach of energy cutoff to identify all points belonging to basins in the energy surface and using a *K*-means clustering approach to collect the points belonging to the same minimum proved to be robust and efficient. Since the size of the basins and thus also the total integrated probability depend on the value of the energy cutoff parameter, we analyzed its convergence behavior. The effect the cutoff has on defining the basins is shown schematically in Figure 3a. To validate the assignment of a point ***r***_∥_ to a basin, we use an additional criterion—the Pt–H height. It turns out that this quantity shows an almost abrupt transition when going from the hollow to the on-top configuration. The (non-normalized) probability to find an adsorbate in basin B is then given by
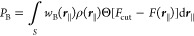
4

Here, the integral goes over the unit cell area *S* and Θ, the Heaviside step function. *w*_B_(***r***_∥_) is a weight function obtained from the *K*-means clustering and tells whether a given point ***r***_∥_ on the surface *S* is part of basin B or not
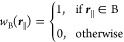
5

The convergence of the free energy difference Δ*F* and the ratios of the probabilities as determined by *F*_cut_ are shown in Figure 3b,c, respectively. Increasing the cutoff *F*_cut_ increases the size of the basins until convergence is reached within a certain cutoff criterion. As cutoff criterion we enforce that the free energy difference between two sites is converged below <1 meV.

### 3.1 Free Energy Differences: Vacuum *vs* Solvent

**Figure 4:**
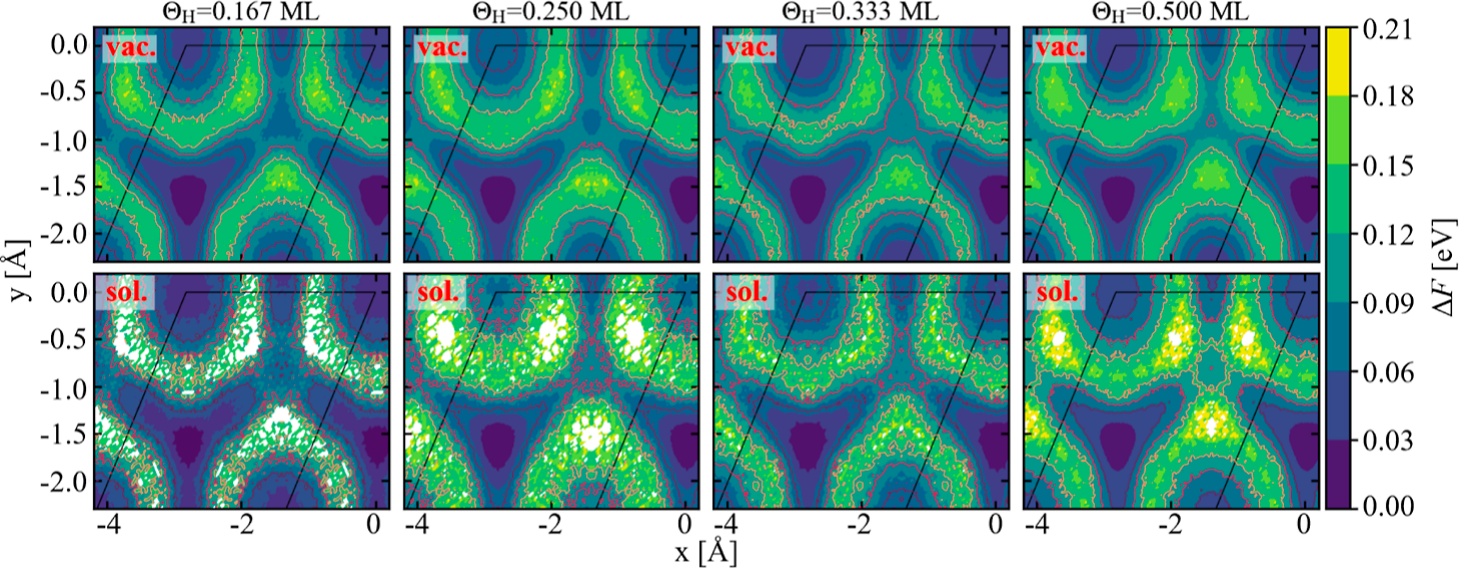
Free energy surface of H adsorbed on the Pt(111) surface in contact with a vacuum (top row) and water (bottom row) for various H coverages. The energy minimum of each surface is set to zero. The energy surface has been constructed from the AIMD simulations using the method described in the text. The trapezoidal black box marks the primitive surface unit cell, which contains a single surface Pt atom. The various high-symmetry adsorption sites are shown in Figure 2a.

**Figure 5:**
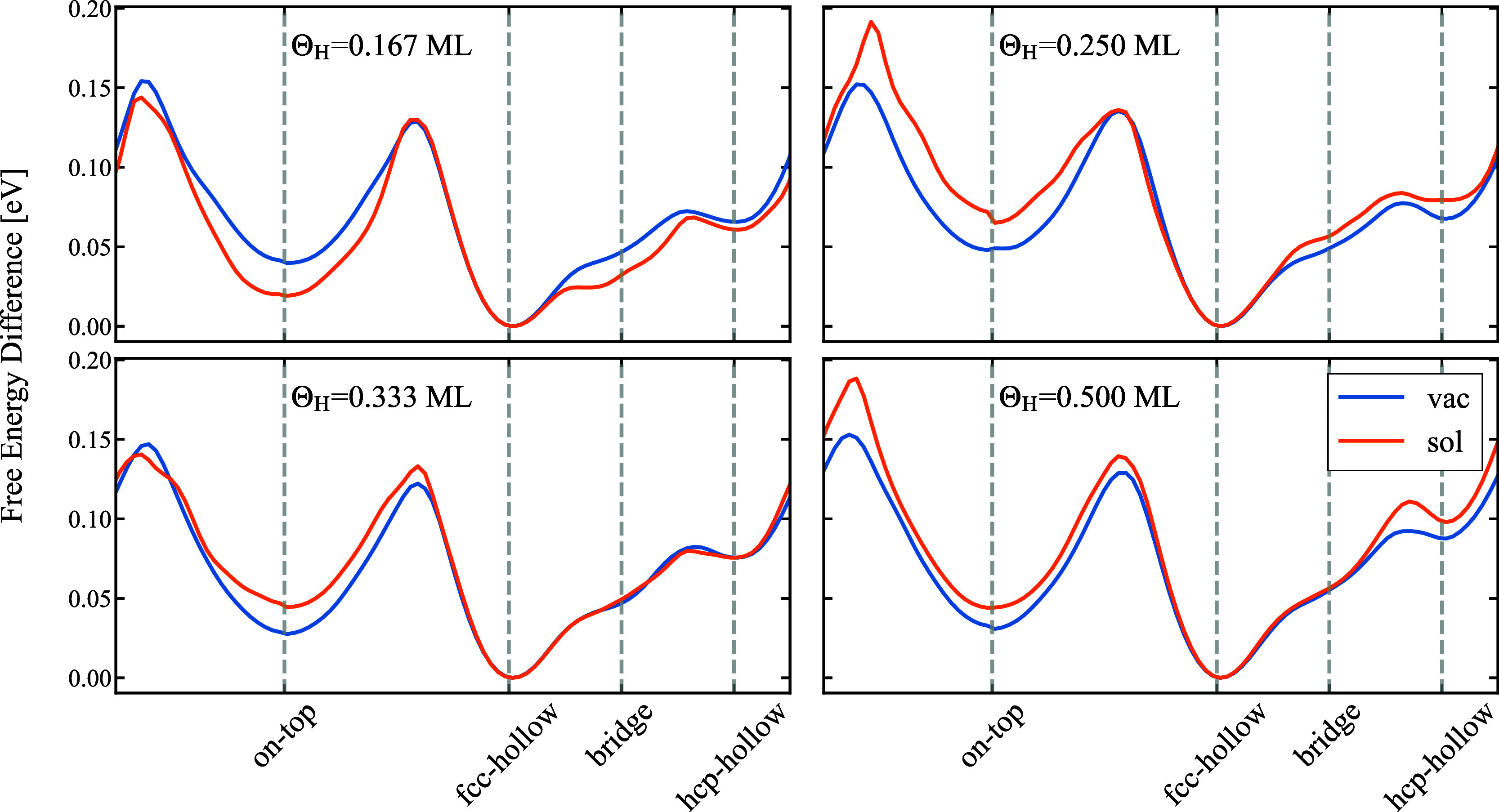
Free energy surface of H adsorbed on Pt(111) along a path connecting the high-symmetry adsorption sites for various H coverages (indicated in each respective plot). The blue/orange lines show results for the vacuum/water interface.

Running the MD simulations described in Section 2 and performing the analysis described in the previous section we obtain the free energy surface for adsorbed H at various coverages and in the presence/absence of water. The results are summarized in Figures 4 and 5. Both figures clearly show that both coverage and presence of water have surprisingly little impact on the free energy surface and thus on the adsorption thermodynamics and kinetics of H on Pt(111). The higher noise levels visible in the presence of water are a consequence of the reduced simulation times (150 ps without water versus 30 ps with water).

For all coverages and the two scenarios (with/without water) the free energy difference between on-top and fcc-hollow site is around (50 ± 10) meV (Figure 5). This energy difference corresponds to roughly twice the thermal energy (2*k*_B_*T*), *i.e.*, a substantial amount of adsorbed H occupies the on-top site even at room temperature. For the Θ_H_ = 1/2 ML without water for instance, this corresponds to an average on-top site occupation probability of ≈25% as seen in Figure 3c.

The close resemblance of the free energy surface for different coverages in the presence/absence of water applies not only to the (local) minima, but also to the free energy surface. It applies to the entire surface and thus also to the kinetic barriers. Having the free energy surfaces, we can directly identify which configurations are (meta-)stable, *i.e.*, have a global (local) minimum, and which are unstable. Both, the fcc-hollow configuration (global minimum) and the on-top one are surrounded by large barriers, thus confining the H adatoms for a substantial time. In contrast, the hcp-hollow site shows only a very shallow local minimum and is also energetically rather high. It therefore does not play any role in the adsorption behavior. The final high symmetry adsorption site, the bridge site, shows no local minimum. Rather, it is contained within the attractive basin of the fcc-hollow site and therefore does not represent a separate adsorption site.

We can also use the 2D free energy surface to identify the minimum energy paths between the (meta)stable adsorption sites. As can be seen in Figure 4 the diffusion path to go from the fcc-hollow to the on-top site is not *via* bridge to hcp-hollow to on-top site. Rather, the lowest energy barrier is realized via a direct jump parallel to the *y*-axis (along [010]). This barrier is ≈10 meV lower than the one shown in Figure 5 going along the diagonal path.

## 4 Computing Laterally Resolved Vibrational Spectra

In addition to the free energies, we also computed the atom resolved spectra for the Pt–H stretch vibrational mode (perpendicular to the electrode surface). Just as for the free energy profiles, the only input required for the computation of laterally resolved spectra are the trajectories on the H adsorbates. Typically, vibrational spectra for these modes are computed from the velocity autocorrelation function.^7^ Here, we take the velocity of the adsorbates along the surface normal direction (using finite differences) and then use this as the “signal” for a fast Fourier transform (FFT) procedure. To compute the “total” vibrational density of states (VDOS) for the adsorbates, we use the Welch periodogram method.^44^,^1^ This method allows us to divide the signal into overlapping segments and then compute the averaged spectral densities using a standard periodogram algorithm. In this case, a segment width of 720 fs is used and a Tukey type windowing function,^45^,^1^ is applied to the signal (see the Supporting Information).

**Figure 6:**
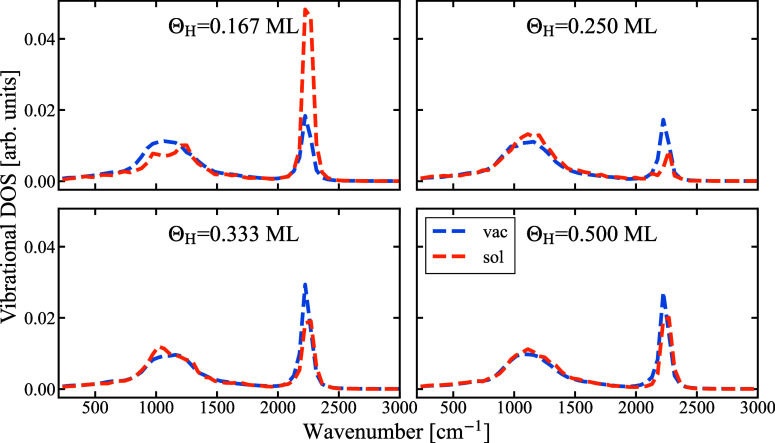
VDOS for the H–Pt(111) stretch mode for various H coverages. The spectra are obtained from MD simulations for an interface with vacuum (blue) and water (orange).

The resulting VDOS for the vacuum and solvent structures and for various coverages are shown in Figure 6. For both the vacuum and solvent structures, we see a broad peak corresponding to 1100 cm^–1^ and a peak at 2200 cm^–1^ which agree well with the ones reported by experimental and theoretical studies of this system.^7,11,29^ As already deduced from the free energy comparison, the presence of H_2_O has no or little impact on the vibrational spectra of these H-adsorbates.

**Figure 7:**
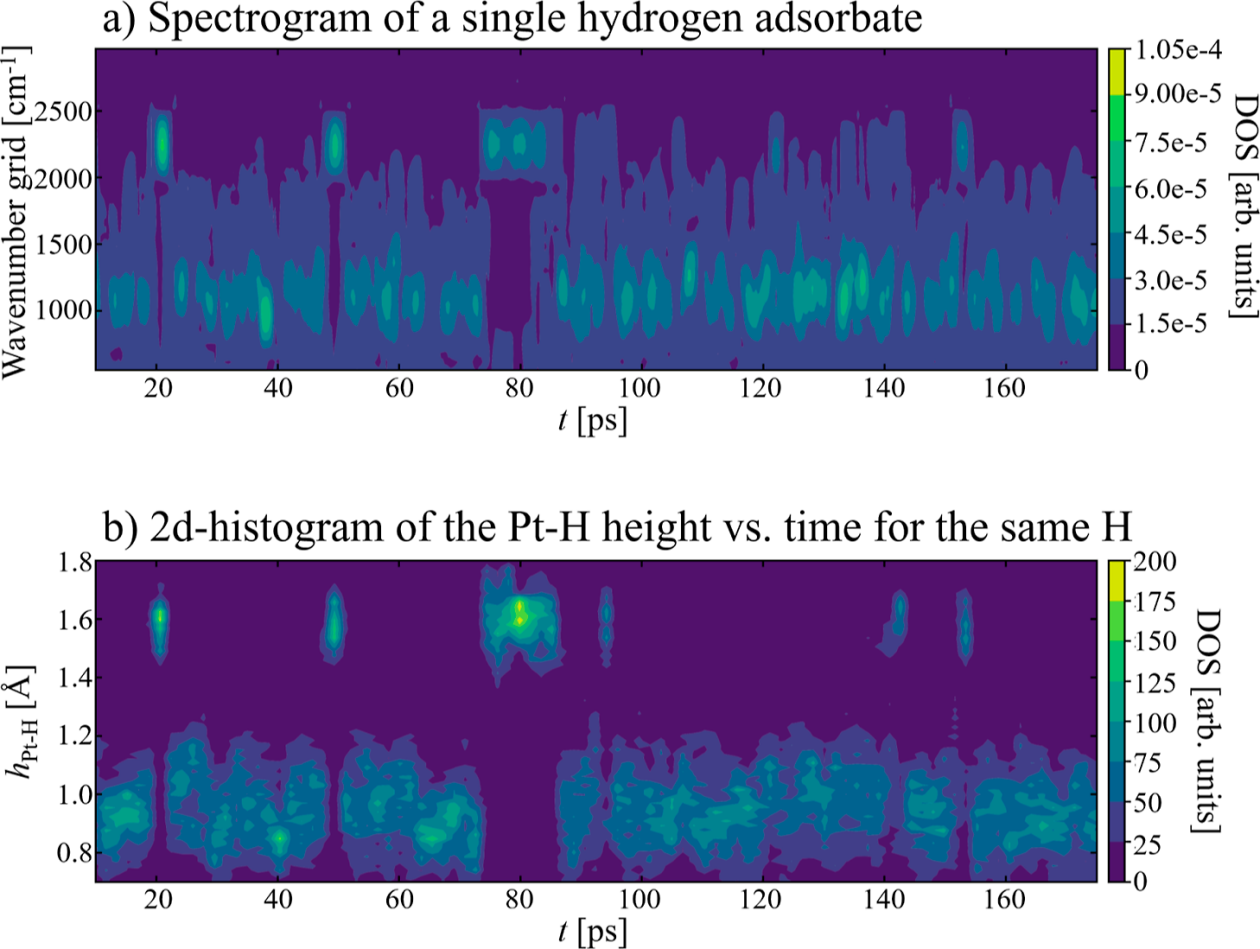
(a) The atom resolved spectrogram *S*_H_^I^(*t*,ω) of a single H adsorbate in the Θ_H_ = 1/2 ML system (without water) and (b) the corresponding two-dimensional histogram showing the evolution of the Pt–H height (along the surface normal direction) for the very same H adsorbate with time. Note the correlation between the adsorbate height (which reflects the site) and frequency for any given snapshot.

To assign the observed peaks to corresponding adsorbate sites, we resolve the spectral intensities for each H adatom as a function of time. From the computed trajectories of each H adatom, we can thus directly correlate the vibrational frequency to this atom and its lateral position at any given time. To determine the time-resolved spectrum of each H adatom, we use the spectrogram method. Spectrograms give the time dependence of the frequency spectra of a given signal by dividing the signal into sequential (but overlapping) segments of a given width.^47^ For each of these segments, the spectrum is calculated. The spectral intensities of these segments are then mapped onto the trajectories of each of the adsorbates within these segments in the following way:

The spectrogram method, when applied to the velocity of the adsorbates along the surface normal direction, returns the time-resolved spectral density. For each hydrogen adsorbate denoted by I_H_ (the index I_H_ runs over the number of H atoms on the surface, starting at 1), we obtain a spectrogram density denoted by *S*_H_^I^(*t*_*s*_,ω) where *t*_*s*_ and ω correspond to time and frequency grid points obtained from the spectrogram. From this quantity, for a given frequency range defined by the two end points ω_1_ and ω_2_, the frequency averaged spectral density for each of these adsorbates at a given DFT-MD snapshot at time *t* is
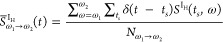
6

 is the number of frequency grid points in that frequency range and δ is the Dirac delta function. For the Θ_H_ = 1/2 ML interface without water, the spectrogram for one such adsorbate is visualized in Figure 7a. When compared with the evolution of the height *h*_Pt–H_ of the H above the Pt layer for the same adsorbate, which is represented as a histogram in Figure 7b, the strong correlation between the two adsorption sites (with the on-top sites having larger *h*_Pt–H_ values than the fcc-hollow sites) and the corresponding change in the spectral frequencies of the atoms is evident. From the time dependent  we get the spatially resolved spectral density as.
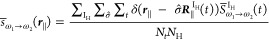
7

Like in eq 1, the lateral positions  of the H adsorbates are represented on a grid spanning the primitive Pt(111) surface unit cell (labeled by ***r***_∥_). The sums run over the number of hydrogen adsorbates *N*_H_, the number of the DFT-MD time steps *N*_*t*_, and the point group and translational symmetry operators of the Pt(111) surface . This quantity, , gives the probability to find a H atom vibrating in the frequency range ω_1_ → ω_2_ on the lateral position ***r***_∥_. This is visualized for various frequency ranges and for all studied interfaces in Figure 7.

For a good spatial resolution of our profiles, the width of the segment should be as small as possible. However, in spectrograms, higher temporal resolution comes at the cost of the frequency resolution, and therefore, an optimum value for the segment width should be chosen such that certain vibrational frequencies (or more accurately: frequency ranges) can be assigned to certain lateral positions of the adsorbate on the surface of the electrode. While a systematic way to choose the width of these segments may be difficult, based on a careful analysis of the resulting spectrograms, a segment width of 540 fs is chosen. Similar to the computation of the total VDOS, a Tukey-type windowing is applied to the signal segments (see the Supporting Information).

**Figure 8:**
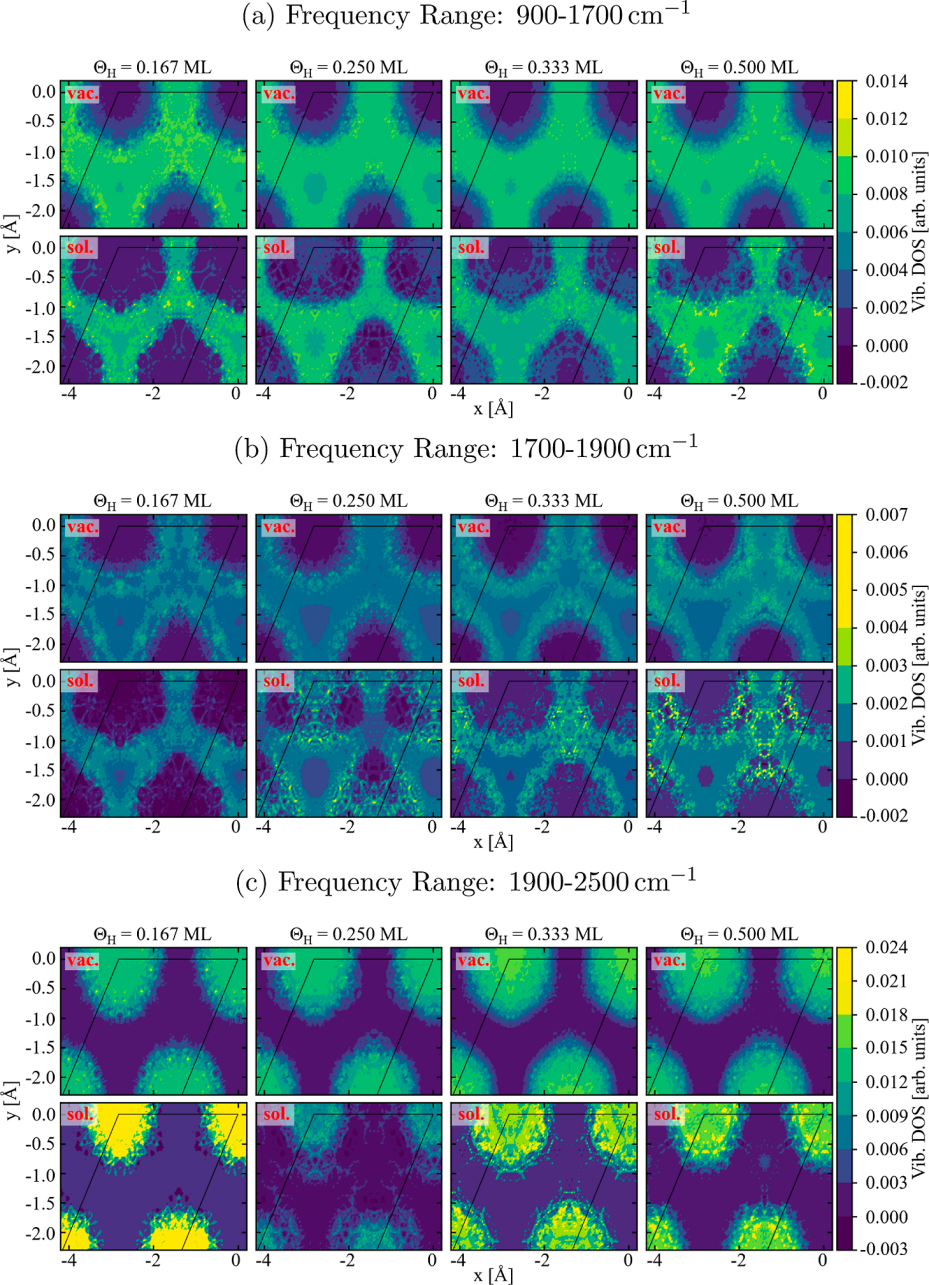
Laterally resolved spectral intensity for the frequency range from 900 to 1700 cm^–1^ (top two rows), from 1700 to 1900 cm^–1^ (two middle rows) and from 1900 to 2500 cm^–1^ (two bottom rows) for H adsorbed on Pt(111) under both vacuum and H_2_O covered surfaces. The color code marks the intensity integrated over the considered frequency range with low intensities (dark blue), intermediate intensities (green), and high ones (yellow).

The laterally resolved profiles of three representative frequency ranges are shown in Figure 8. The Figure 8a,b show the lateral positions of H (yellow and green colors) in the frequency interval of the first and rather broad peak in Figure 6 (900–1700 cm^–1^). Interestingly, this frequency window is not restricted to the hollow site but is found for all adsorbate configurations except the ones related to the on-top position. The bottom rows, *i.e.*, Figure 8c, show the frequency interval of the second and sharper peak in Figure 6. As can be seen, the sources of the second peak are exclusively adsorbate positions corresponding to the on-top site.

Comparing the maps for the first and second peaks (top and bottom rows), we find that they are practically exclusive: The second peak originates exclusively from on-top configurations, while the first one arises from all other configurations. It is interesting to note that the first peak cannot be exclusively assigned to the most stable fcc-hollow site but also appears for energetically less favorable positions such as the bridge or hcp-hollow sites. Thus, while the second peak provides a clear and unique spectral fingerprint for H being in the on-top configuration the lower peak provides only information that the H is *not* occupying the on-top position.

Having this relation between the spectral intensity and lateral adsorbate position allows us to study whether specific spectral regions can be identified as being particularly sensitive to certain configurations. That this type of selectivity exists is shown in the two middle rows of Figure 8, which show the frequency region between the two main peaks, *i.e.*, from 1700 to 1900 cm^–1^. In this region, the highest intensity is observed for configurations that are far away from any of the high-symmetry adsorption sites. This finding is thus a clear indication that spectra can be used not only to probe the presence of atoms in specific stable or metastable configurations. Rather, also intermediate short-lived configurations, resulting *e.g.* from thermal excitations, may have a pronounced spectral signature. Such spectral signatures provide the unique opportunity to study intermediate configurations experimentally.

A critical condition to finding such states is that the free energy surface must be rather shallow. This shallowness allows H adatoms to explore regions that are far away from the conventionally considered adsorption sites. This large lateral distribution also explains why the first peak is so broad: Its source is not a single spatially well localized site (as the on-top site shown in the two bottom rows), but it originates from several structurally rather different adsorbate configurations.

The results with water are more noisy, which is again a consequence of the shorter affordable simulation times and the fluctuations induced by the highly mobile water molecules. However, the coverage dependence and the presence of water again have little effect on the laterally resolved spectra.

The reason why water has only such a small impact on the laterally resolved H adsorption spectra and free energy surface is a direct consequence of the much stronger bond formed between H and Pt, compared to the interaction of H with water. The weak interaction of adsorbed H with water follows directly from the large hydrophobic gap. As shown in ref (6), adsorbed H repels adsorbed water molecules on the neighboring sites. Thus, the presence of H further widens the hydrophobic gap in its vicinity, leading to an additional decrease in the level of interaction between water and adsorbed H.

## 5 Conclusions

Exploiting translational and rotational surface symmetry, we were able to boost the statistical convergence of spatially resolved quantities such as adsorbate free energies or vibrational frequencies. With this approach, it became possible to accurately and computationally highly efficiently map out the free energy surface and the H–Pt vibrational spectrum onto the lateral position of the adsorbed H atoms using ab initio MD runs as short as 20 ps. Employing this approach, we have systematically analyzed the impact of water and coverage on the adsorption behavior of H on Pt(111). A key insight of this study is that for H adsorption, the impact of water on free energy differences and spectra can be largely neglected. We note that this insensitivity to the presence of water does not apply universally to all adsorbate related properties of H: In a recent study^6^ we showed that the presence of water qualitatively impacts properties highly relevant for electrochemical reactions, such as the electrode potential or the onset of H desorption.

The spatial resolution of the vibrational H-related modes achieved in this study provides fundamental insight into which adsorbate configurations can be spatially probed. The on-top configuration is connected to a high-frequency peak at ≈2200 cm^–1^, which is in line with previous assignments of this peak. For the lower and very broad mode we show that it does not result from a pure single adsorption site. Rather, it is a superposition of all adsorbate configurations except ones related to the on-top position. Its origin is a shallow and highly anharmonic free energy surface basin centered around the fcc-hollow site. Our study showed that spectral fingerprinting is not limited to stable and metastable configurations but may even capture intermediate configurations. The proposed approaches to efficiently compute spatially resolved properties, such as adsorbate free energies or frequencies, is generic and can be straightforwardly applied to a wide range of adsorption phenomena.

## Footnotes

1 As implemented in the Scipy^46^ python package modules for signal processing https://docs.scipy.org/doc/scipy/reference/signal.html.
