# Supplementary material for: Laterally Resolved Free Energy Profiles and Vibrational Spectra of Chemisorbed H Atoms on Pt(111)
Source: J Chem Theory Comput. 2024 Feb 7;20(5):2192–201. doi: 10.1021/acs.jctc.3c00997 (PMC10938496; doi:10.1021/acs.jctc.3c00997)
Supplement: Supplementary file 1 — ct3c00997_si_001.pdf [file ct3c00997_si_001.pdf]

Supplementary Information: Laterally Resolved Free Energy Profiles and Vibrational Spectra of Chemisorbed H Atoms on Pt$(1\,1\,1)$

Sudarsan Surendralal, Mira Todorova,* and Jörg Neugebauer

Department of Computational Materials Design, Max-Planck-Institut für Eisenforschung GmbH, Max-Planck-Straße 1, D-40237 Düsseldorf, Germany

E-mail: m.todorova@mpie.de

Computational details of calculating the spectra

The Welch[1] method obtains the power density spectra of a given signal in time domain by dividing the signal into overlapping segments, applying a periodic window function to these segments, and computing the FFT on each of these segments to obtain the spectral density of the signal segment (i.e., the "periodogram"). The periodograms of these individual segments are then averaged to get the total vibrational spectra. Averaging over the segments helps to reduce the statistical variance in the spectra, compared to taking the FFT of the entire signal at once. Window functions are used to avoid discontinuities in the spectra which occur when FFT is applied to signal segments which are finite and non-periodic (FFT is intended for periodic signals). On multiplying the window function with the individual segments, continuity and periodicity of the segments is ensured by making the signal segment "taper-off" to zero at the edges (i.e. the beginning and end of the signal segment). Many choices of window functions exist mainly differing by how the multiplied signal segments taper off at

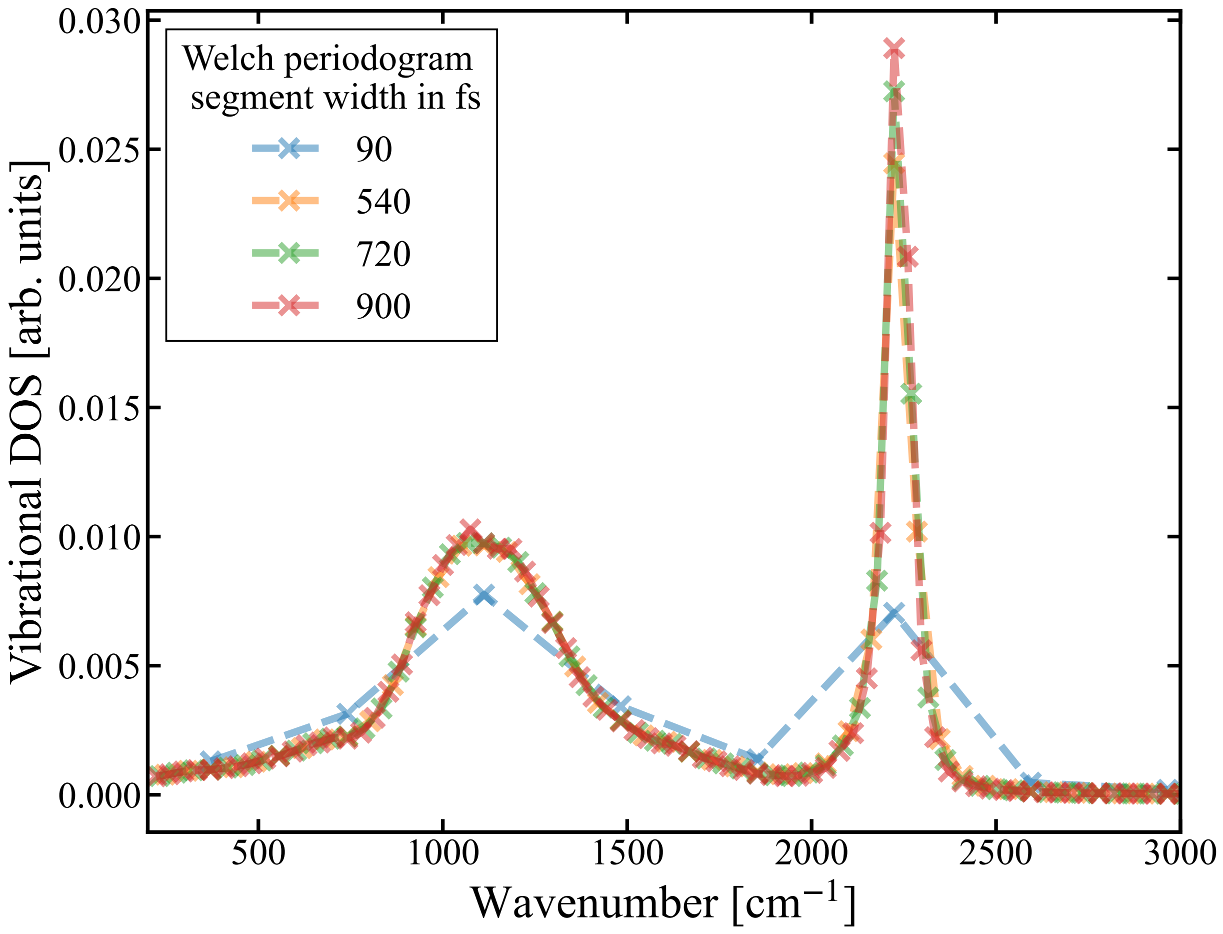

Figure S1: Vibrational density of states for the H-Pt(111) stretch mode for the $\Theta_H = 1/2\,\mathrm{ML}$ interface without water for different choices of the periodogram segment width.

the edges. We chose the popular "Tukey" window function[2] where a cosine based tapering of the edges is used. The overlap width is determined by a shape parameter which determines which fraction of the segment is tapered off on either side of the signal.[3]

The choice of the segment width for the Welch method has to be carefully chosen. On the one hand, a large segment width ensures a finer frequency resolution, which is necessary to capture the essential features of the spectra. On the other hand, a large segment width results in fewer segments to average over, which in turn, results in larger statistical variance in the averaged spectra. The effect of the segment width on frequency resolution is evident in Fig. S1 where the vibrational spectra for the $\Theta_H = 1/2\,\mathrm{ML}$ interface without any solvent is plotted for different choices for the segment width. While the frequency resolution

of the vibrational density of states with a segment width of 90 fs is too coarse to capture essential features of the spectra, those obtained using widths of 540 fs and above give better converged results. For the total vibrational density of states, a segment width of 720 fs is chosen as a compromise between spectral resolution and statistical convergence. The used Tukey window overlap width of 25% of the segment width corresponds to 180 fs.

The spectrogram method, which gives the time resolved spectra, also requires a segment width as an input. Time resolution is achieved at the cost of frequency resolution, as mentioned in the main text. Therefore, since time resolution is also critical, an even smaller segment width needs to be chosen. For the spectrograms, a segment width of 540 fs which as seen in Fig S1, still captures the essential features of the spectra, is chosen. The corresponding 25% Tukey window overlap width is 135 fs.

References

(1) Welch, P. The use of fast Fourier transform for the estimation of power spectra: A method based on time averaging over short, modified periodograms. *IEEE Transactions on Audio and Electroacoustics* **1967**, *15*, 70–73.

(2) Harris, F. On the use of windows for harmonic analysis with the discrete Fourier transform. *Proceedings of the IEEE* **1978**, *66*, 51–83.

(3) Virtanen, P. et al. SciPy 1.0: Fundamental Algorithms for Scientific Computing in Python. *Nature Methods* **2020**, *17*, 261–272.
